# 606 Far Out: Evaluating the Impact of Distance on Acute Burn Outcomes

**DOI:** 10.1093/jbcr/iraf019.235

**Published:** 2025-04-01

**Authors:** Alexis Henderson, Hilary Liu, José Arellano, Christopher Fedor, Mare Kaulakis, Garth Elias, Alain Corcos, Jenny Ziembicki, Francesco Egro

**Affiliations:** University of Pittsburgh School of Medicine; University of Pittsburgh Medical Center; University of Pittsburgh Medical Center; University of Pittsburgh School of Medicine; University of Pittsburgh School of Medicine; University of Pittsburgh Medical Center; University of Pittsburgh Medical Center; University of Pittsburgh Medical Center; University of Pittsburgh Medical Center

## Abstract

**Introduction:**

Burn centers are disproportionately concentrated in urban areas, threatening to produce health disparities among rural patients. Delays in treatment can lead to longer hospital stays, higher risk of complications, delayed wound healing, and increased mortality. This study aims to determine the impact of patients’ distance to a single burn center on burn outcomes.

**Methods:**

A 5-year retrospective review was conducted on patients who presented to a single ABA-verified burn center with acute burn injuries between 2016 and 2023. Data collection included demographics, distance to burn center, burn characteristics, and outcomes. Multiple logistic regression and analysis of variance were performed to analyze the effect of distance on burn outcomes.

**Results:**

A total of 2777 patients (mean age 41.1 ± 24.4 years; 81.4% Caucasian, 13.7% African American, 2.23% Native Hawaiian/Pacific Islander, 0.97% American Indian/Alaska Native) were included. The average distance from home to the burn center was 146.0 ± 745.2 km, and time from injury to admission was 1.04 ± 2.9 days. Ventilator use averaged 0.8 ± 4.0 days, ICU stay 3.2 ± 9.0 days, and hospital stay 9.1 ± 12.15 days, with an average of 0.81 ± 1.5 operations. Most patients (71.7%, n=1992) had 2nd or 3rd degree burns, with a mean TBSA of 7.3 ± 12.4%. Native Hawaiians/Pacific Islanders lived the farthest (386.6 ± 1624.1 km), and African Americans the closest (99.9 ± 688.1 km).

Distance from the burn center was not significantly associated with mortality (χ²=4.32; p=0.825), ventilator days (β=0.234; p=0.968), operations (β=0.822; p=0.966), TBSA (β=0.210; p=0.899), hospital days (β=-1.831; p=0.608), or ICU days (β=-0.165; p=0.959). However, longer delays between injury and admission were significantly associated with greater distance (β=15.20; p=0.0068). Significant differences in distance were found between racial groups (χ²=268.3; p< 0.001).

**Conclusions:**

Patients living far from a burn center are more likely to have a delay between their date of injury and the date of admission than patients living closer, and distances vary significantly between racial groups. Interestingly, distance does not have a significant effect on patient mortality and length of hospital stay.

**Applicability of Research to Practice:**

The significant findings outlined here point to potential inequities associated with delays in treatment. Efforts in this area should be geared towards telemedicine for remote consultations and more robust community education on the treatment of acute burns, especially for those without access to care.

**Funding for the Study:**

N/A

